# Structure of Randomly Distributed Nanochain Aggregates on Silicon Substrates: Modeling and Optical Absorption Characteristics

**DOI:** 10.3390/ma15144778

**Published:** 2022-07-07

**Authors:** Tianze Zhao, Yanze Gao, Rui Shi, Zhuo Li, Qingfeng Shi

**Affiliations:** 1 School of Optics and Photonics, Beijing Institute of Technology, Zhongguancun South Street 5, Beijing 100081, China; zhaotz12@163.com (T.Z.); bitsr@126.com (R.S.); lizhuo@bit.edu.cn (Z.L.); 81908027@bit.edu.cn (Q.S.); 2 Beijing Key Laboratory for Precision Optoelectronic Measurement Instrument and Technology, Zhongguancun South Street 5, Beijing 100081, China; 3 Analysis & Testing Center, Beijing Institute of Technology, Zhongguancun South Street 5, Beijing 100081, China

**Keywords:** optical absorber, nanoparticle clusters, morphological reconstruction, equivalent physical characteristics, visible-range absorption

## Abstract

Nanoparticle aggregate structures allow for efficient photon capture, and thus exhibit excellent optical absorption properties. In this study, a model of randomly distributed nanochain aggregates on silicon substrates is developed and analyzed. The Gaussian, uniform, and Cauchy spatial distribution functions are used to characterize the aggregate forms of the nanochains and their morphologies are realistically reconstructed. The relationships between the structural parameters (thickness and filling factor), equivalent physical parameters (density, heat capacity, and thermal conductivity), and visible absorptivity of the structures are established and analyzed. All the above-mentioned parameters exhibit extreme values, which maximize the visible-range absorption; these values are determined by the material properties and nanochain aggregate structure. Finally, Al nanochain aggregate samples are fabricated on Si substrates by reducing the kinetic energy of the metal vapor during deposition. The spectral reflection characteristics of the samples are studied experimentally. The Spearman correlation coefficients for the calculated spectral absorption curves and those measured experimentally are higher than 0.82, thus confirming that the model is accurate. The relative errors between the calculated visible-range absorptivities and the measured data are less than 0.3%, further confirming the accuracy of the model.

## 1. Introduction

Nanostructures consisting of metal nanoparticle clusters can be described as aggregates of nanocells. These sparse and porous nanostructures can efficiently capture photons, and thus exhibit excellent optical properties [1,2,3]. Hence, optical absorbers based on nanoaggregate structures are widely used in microbolometers [4], photothermal converters [5,6], solar cells [7,8], and photocatalysis [9]. In addition, nanoaggregate structures show several desirable physical properties, such as low heat capacity, high specific surface area, and low density. As a result, these structures have shown significant potential in the fields of tunable excitation radiation and photothermoacoustics [10].

Several quasianalytical models have been developed for characterizing the nanoaggregate structures, including the effective medium model [11], cluster-cluster aggregation (CCA) model [12], and fractal lossy antennas (FLA) model [13]. The effective medium model simplifies complex nanoclusters into hypothetical ellipsoids. However, the permittivity elucidated by the effective medium model is based on statistical parameters, and the model lacks a description of the nanostructures. The CCA model represents the aggregate morphology of spherical units and the growth process of clusters based on the theory of diffusion-limited aggregation [14,15,16]. The FLA model describes the nanoaggregate structure as a forest-like structure composed of chains. The length of the chains in the FLA model is the order of microns.

In this paper, a new model of randomly distributed nanochain aggregate structures is proposed to describe their micromorphology. The relationships between the structural parameters (thickness and filling factor), equivalent physical parameters (density, heat capacity, and thermal conductivity), and visible-range absorptivity of the model were established and analyzed. These relationships can serve as a theoretical reference for further research on areas such as the study of the photothermoacoustic effect of nanoaggregate structures. To evaluate the model, Al nanochain aggregate structures were fabricated on Si substrates, and the spectral reflection characteristics of the samples were determined experimentally. The experimental results showed that the model can realistically reconstruct the morphology of the structures and allows for accurate calculations of their spectral absorptivity.

## 2. Materials and Methods

### 2.1. Fabrication of Nanoaggregate Structures

**Figure 1:**
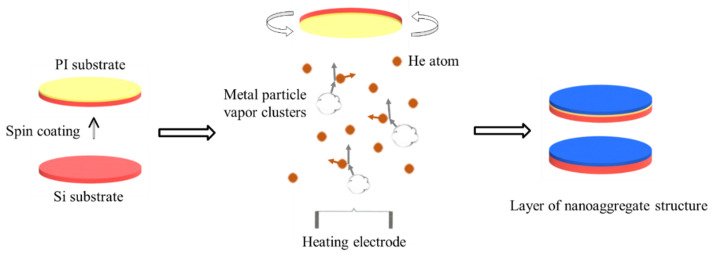
Process for fabricating layer of nanoaggregate structure.

To fabricate nanoaggregate samples, we chose two types of substrates, Si wafers and polyimide (PI) films. The Si wafers were cleaned using an oxygen plasma. The PI films were fabricated on Si wafers by spin coating. A 6 mL polyamic acid (PAA) solution was spin-coated on the Si wafers at a low speed (800 r min^−1^) for 1 min and then at a high speed (7000 r min^−1^) for 3 min. The PAA coating was then air-dried at 25 °C for 1 h and cured in an oven filled with N_2_ gas for 3 h at 300 °C to chemically transform PAA into PI. The process for fabricating a layer of the nanoaggregate structure is shown in Figure 1. Metal Al was evaporated in a He atmosphere at a pressure of 980 Pa, and the Al metal vapor was made to pass through the He atmosphere to reduce its kinetic energy before it was deposited on the substrate.

### 2.2. Model of Nanochain Aggregates

#### 2.2.1. Basic Structural Unit of Model

**Figure 2:**
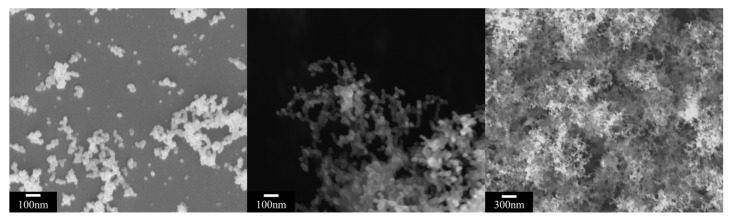
SEM images of nanoaggregate structure.

We obtained scanning electron microscopy (SEM, Supra55, Zeiss, Oberkochen, Germany) images of the fluffy, “smoke-like” metal deposition structure, as shown in Figure 2. The fluffy structure is porous, micron sized, and composed of a large number of clusters, which, in turn, consist of randomly distributed nanochains. These nanochains are composed of metal nanoparticles, which can be considered as nanospheres.

#### 2.2.2. Spatial Distribution of Nanochains

**Figure 3:**
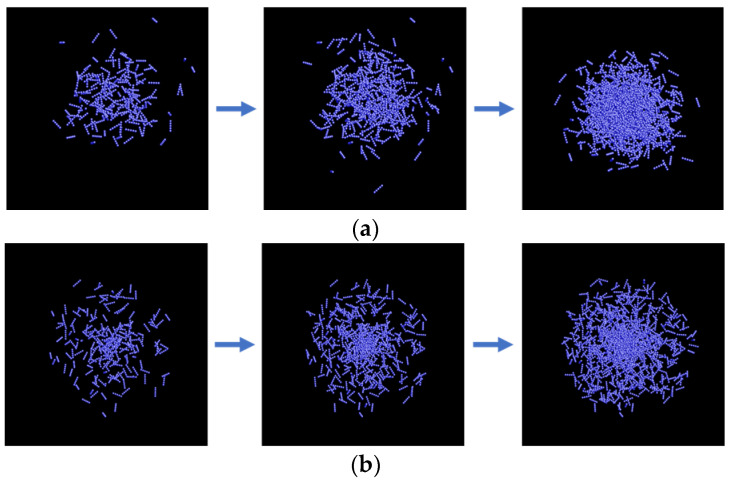

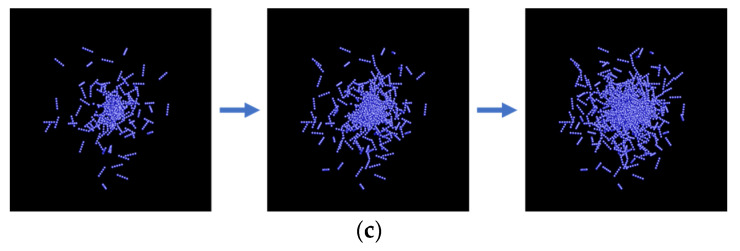
Nanochain cluster models generated using different distribution functions. (**a**) Clusters whose form is represented by Gaussian distribution. (**b**) Clusters whose form is represented by uniform distribution. (**c**) Clusters whose form is represented by Cauchy distribution.

A large number of nanochains were generated in a three-dimensional space to form clusters. The distribution of nanochains is disordered, but this disordered distribution shows statistical regularity. Therefore, we try to use classical probability distribution functions, Gaussian, uniform and Cauchy distributions, to characterize such statistical regularity. The spatial distribution function represents the aggregate form of the nanochains and reflects the probability density distribution of the nanochains in the clusters, as shown in Figure 3. In the spatial coordinate system with the cluster centroid as the origin, the probability density functions corresponding to the three spatial distributions are as follows:

(1) $$G(r)=\frac{1}{\sqrt{2\pi }\;\sigma }\exp(-\frac{r^{2}}{2\sigma^{2}}),$$

(2) $$U(r)=\frac{1}{2\sigma \;\sqrt{3}},$$

(3) $$C(r)=\frac{1}{\pi }(\frac{\sigma }{r^{2}+\sigma^{2}}),$$

where G(*r*), U(*r*), and C(*r*) represent the probability density functions of the Gaussian, uniform, and Cauchy distributions, respectively; *r* represents the distance between the nanochain and the cluster centroid; *σ* is the size parameter of the spatial distribution, which determines the radius of the cluster.

As a structural parameter, the distribution function used affects the propagation path of photons within the structure. Thus, the selection of the appropriate distribution function is essential for accurately calculating the optical absorption. Given that the actual spatial distribution of nanochains is highly random, we add a weight to each distribution function to improve the accuracy of the model, demonstrated by the following equation:

(4) $$W_{G}+W_{U}+W_{C}=1,$$

where *W_G_* is the weight of the nanochain clusters based on the Gaussian distribution function; *W_U_* is the weight of the nanochain clusters based on the uniform distribution function, and *W_C_* is the weight of the nanochain clusters based on the Cauchy distribution function. The weight of the distribution function reflects the number (percentage) of clusters aggregated by a specific distribution function to all clusters in the model.

#### 2.2.3. Aggregation Model of Clusters

**Figure 4:**
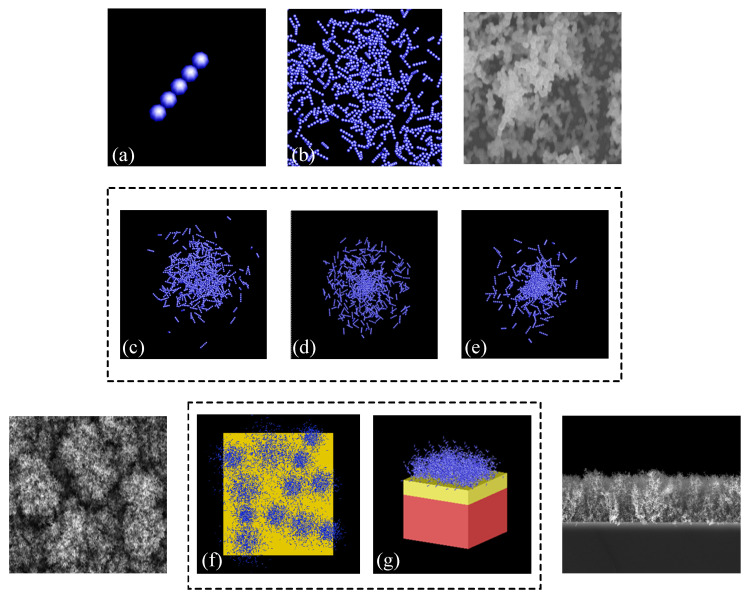
Modeling of nanochain aggregate structure. (**a**) Basic nanochain structure. (**b**) Nanochains with random three-dimensional orientations. (**c**) Cluster based on Gaussian distribution. (**d**) Cluster based on uniform distribution. (**e**) Cluster based on Cauchy distribution. (**f**) Top view of physical model. (**g**) Sectional view of physical model.

In addition to the spatial distribution of nanochains in a cluster, we use the uniform distribution function to describe the spatial distribution of the clusters in the aggregation model as well. Similar to the spatial distribution of nanochains, the spatial distribution of the clusters in the aggregation model can be described as $U(x,y,z)=\frac{1}{2\sigma \;\sqrt{3}}$. This completes the physical modeling of the nanochain aggregate structure, as shown in Figure 4.

We define the dimension of the model in the direction perpendicular to the substrate as the model thickness, *d*. In addition to the thickness, we define a structural parameter, namely, the filling factor (*γ*), to describe the relative density of the nanochain aggregate structure. The filling factor was calculated as the ratio of the structural density to the material density. It also reflects the porosity of the nanochain aggregate structure. In the proposed model, the filling factor was varied by changing the number of nanospheres contained in each cluster.

#### 2.2.4. Finite-Difference Time-Domain (FDTD) Method

The FDTD method was used to calculate the spectral absorptivity of the model [17,18,19,20]. The FDTD method discretized the time-domain Maxwell’s equations by central difference approximation of the spatial and temporal partial derivatives. The finite difference equations with the following form were obtained:

(5) $$\begin{array}{l} E_{x}^{n+1}(i+1,j,k)=\frac{1-\frac{\sigma (i+1/2,j,k)\Delta t}{2\varepsilon (i+1/2,j,k)}}{1+\frac{\sigma (i+1/2,j,k)\Delta t}{2\varepsilon (i+1/2,j,k)}}\cdot E_{x}^{n}(i+1/2,j,k)+\frac{\Delta t}{\varepsilon (i+1/2,j,k)}\cdot \\ \frac{1}{1+\frac{\sigma (i+1/2,j,k)\Delta t}{2\varepsilon (i+1/2,j,k)}}\cdot [\frac{H_{z}^{n+1/2}(i+1/2,j,k)-H_{z}^{n+1/2}(i+1/2,j-1/2,k)}{\Delta y}+\\ \frac{H_{y}^{n+1/2}(i+1/2,j,k-1/2)-H_{y}^{n+1/2}(i+1/2,j,k+1/2)}{\Delta z}] \end{array}$$

where *i*, *j*, and *k* are the grid numbers in *x*, *y*, and z directions, respectively; *n* is the number of time domain iterations; ∆*t* is the time step; *σ* is the conductivity; and *ε* is the dielectric constant. The dielectric constant and conductivity were wavelength-dependent parameters taken from the literature [21]. The reflectivity and transmittivity of the model were calculated using frequency domain power monitors with the following formula:

(6) T(f)=Re(P(f))⋅dS→Re(Psource(f))⋅dS→

where T(f) is the normalized transmittivity of the monitor; P(f) and $P_{source}(f)$ are the Poynting vectors on the surfaces of the monitor and light source, respectively; and $d\vec{S}$ is the differential element of normal direction. The incident light source was set as a plane wave with a wavelength step of 2 nm. To simplify the model and allow for faster computations, the periodic boundary condition was used for the FDTD calculations. The spectral reflectivity (*R_λ_*) and transmittance (*T_λ_*) of the model were obtained using power monitors. The spectral absorptivity (*A_λ_*) of the model was calculated using Equation (7), and the visible absorptivity (*A*) of the model was calculated by integrating the spectral absorptivity.

(7) $$A_{\lambda }=1-R_{\lambda }-T_{\lambda },$$

We designed the Gaussian, uniform, and Cauchy models based on the spatial distribution of the nanochains. The spatial distribution of the clusters within the computational region was uniform for all three models, as described in the previous subsection.

## 3. Results and Discussion

### 3.1. Optical Absorption Properties of Model

#### 3.1.1. Relationship between Filling Factor and Optical Absorption

**Figure 5:**
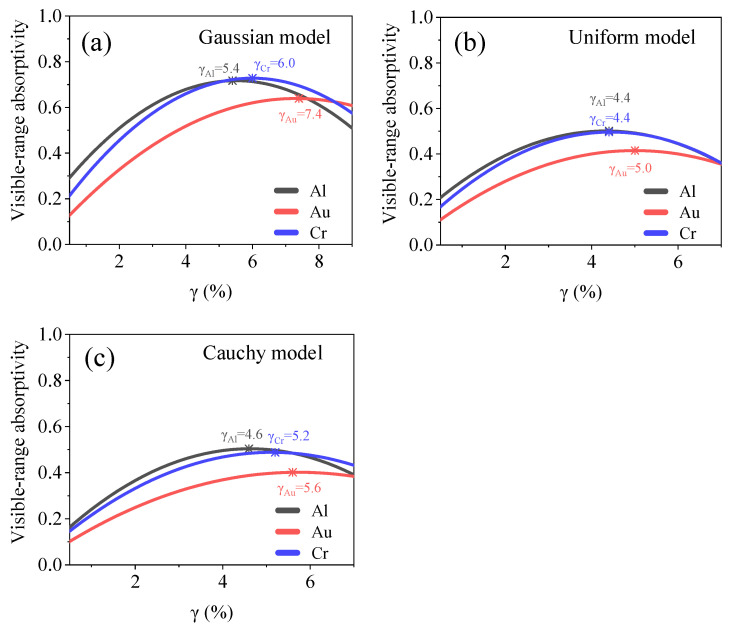
Relationship between optical absorption and filling factor for (**a**) Gaussian, (**b**) uniform, and (**c**) Cauchy models. Filling factor of each model and maximum absorptivity are shown in the figures.

We first analyzed the relationship between the filling factor and optical absorption. We set the nanochain materials as Al, Au, and Cr and used a model thickness of 1 μm and filling factor of 0.5–8%. The spectral absorption curves in the wavelength range of 400–800 nm, as well as the visible-range absorptivity of the model for different filling factors, are shown in Figure 4. The filling factor affects the motion path of the incident photons, and thus the efficiency of the model in capturing photons. An extremely sparse or dense distribution of nanochains leads to a reduction in the model absorptivity. For all three models, the filling factor and visible-range absorptivity exhibited a quadratic relationship. The extreme values of the filling factor, which enhanced the visible-range absorption, are listed in Table 1. We propose the following equation to describe the relationship between the filling factor and visible-range absorptivity:

(8) $$A=c+c_{1}\;\cdot \;\gamma +c_{2}\;\cdot \;\gamma^{2},$$

where *c*_1_, *c*_2_, and *c* are constant coefficients, whose values are determined from the FDTD results. The aggregate form of the nanochains has a significant effect on the visible-range absorptivity, as shown in Figure 5. The Gaussian model possesses the highest visible-range absorptivity, indicating that the nanostructure corresponding to this model is more efficient at capturing photons.

#### 3.1.2. Relationship between Thickness and Optical Absorption

**Figure 6:**
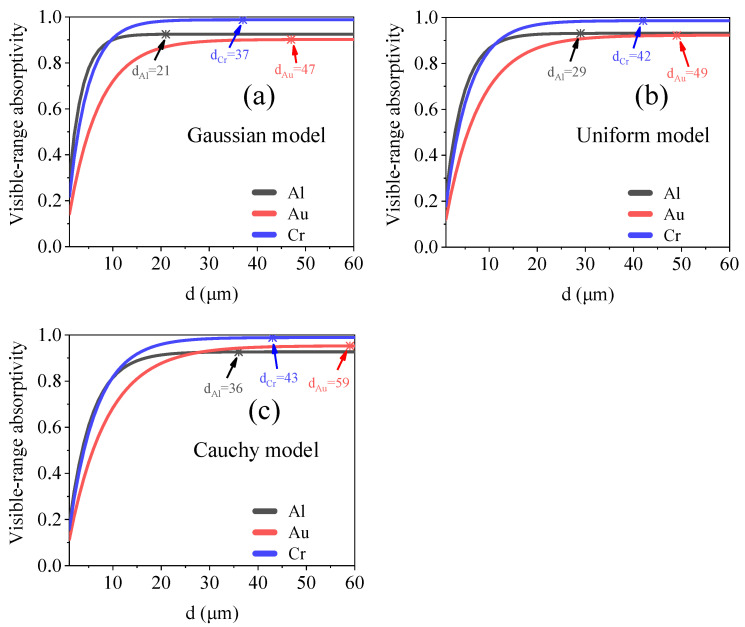
Relationship between optical absorption and thickness for (**a**) Gaussian, (**b**) uniform, and (**c**) Cauchy models. Critical thicknesses of models are shown in the figures.

The nanoaggregate structure demonstrates ultralow surface reflection in the visible band [1,2,3]. For nanochain aggregate structures with the same filling factor, a higher value of thickness would mean that the expected motion path of the incident photons would be longer. Once the thickness of the model exceeds a threshold value, the visible-range transmittance of the model decreases to zero. When the model thickness is more than this threshold value, it no longer affects the visible-range absorptivity of the model. Therefore, the relationship between the thickness and absorptivity takes the form of an exponential function, as shown in Figure 6. We set the nanochain material as Al, Au, and Cr and used the model filling factor of 0.6% and thickness of 1–60 μm. The extreme values of the thickness, which enhanced the visible-range absorption, are listed in Table 1. The critical thickness for the Gaussian model is much smaller than those for the uniform and Cauchy models because of the higher photon-capturing efficiency of the former. The maximum visible-range absorptivities of the three models are similar, indicating that the aggregate form of the nanochains has a negligible effect on the surface reflection of the model. Based on Equation (6), we obtained the following fitting equation:

(9) $$A=c+C_{1}\;\cdot \;(\gamma +C_{2}\;\cdot \;\gamma^{2})\;\cdot \;e^{-d\;/\;C_{3}},$$

where *C*_1_, *C*_2_, and *C*_3_ are constant coefficients, whose values can be obtained from the FDTD results. Equation (7) represents the relationship between the visible-range absorptivity, filling factor, and model thickness. It can be observed that $c_{1}=C_{1}\;\cdot \;e^{-d\;/\;C_{3}}$ and $c_{2}=C_{1}\;\cdot \;C_{2}\;\cdot \;e^{-d\;/\;C_{3}}$.

#### 3.1.3. Relationship between Equivalent Density and Optical Absorption

High optical absorption can be achieved by fabricating nanochain aggregate structures from various materials. Specific application scenarios would result in limitations in terms of the density of the optical absorber. Therefore, it is essential to study the relationship between the equivalent density and optical absorption of nanochain aggregate structures. Based on the above-stated definition of the filling factor, the relationship between the filling factor and equivalent density of the structure can be described as follows:

(10) $$\rho_{eff}=\rho_{0}\;\cdot \;\gamma +\rho_{m}\;\cdot \;(1-\gamma ),$$

where *ρ_eff_* is the equivalent density of the structure; *ρ*_0_ is the density of the material used, and *ρ_m_* is the density of the medium. The structural equivalent densities are affected by the filling factor and material density, as shown in Figure 6a. For vacuum, *ρ_m_* = 0. Therefore, the relationship between *ρ_eff_* and *A* is as follows:

(11) $$A=c+C_{1}\;\cdot \;[\frac{\rho_{eff}}{\rho_{0}}+C_{2}\;\cdot \;{\left(\frac{\rho_{eff}}{\rho_{0}}\right)}^{2}]\;\cdot \;e^{-d\;/\;C_{3}},$$

**Figure 7:**
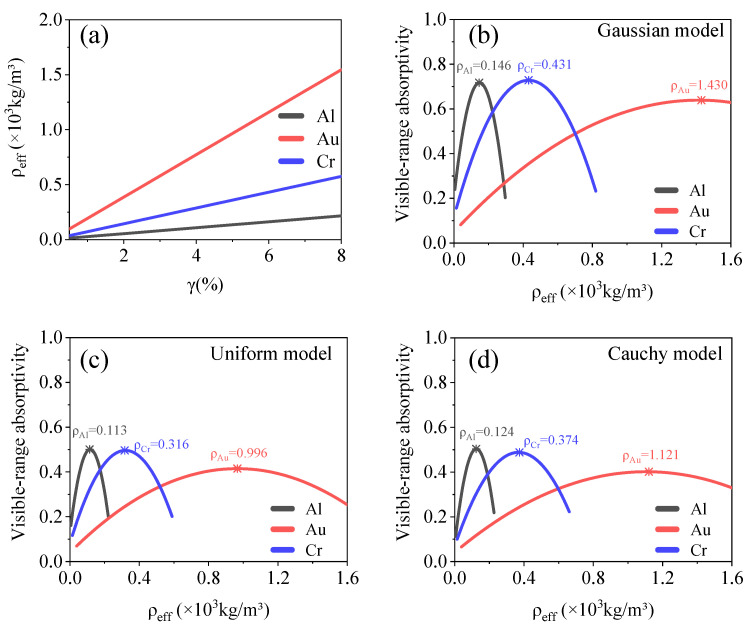
(**a**) Relationship between filling factor and equivalent density of model used. (**b**–**d**) Relationship between equivalent density and visible absorptivity for Gaussian, uniform, and Cauchy models. Equivalent density of model with maximum absorptivity is marked in the figures.

We set the model thickness as 1 μm and the filling factor as 0.6–8%. The nanochain materials included Al, Au, and Cr. The relationship between the equivalent density and visible-range absorptivity of the nanochain aggregate models is shown in Figure 7. The models use the same structural parameters. Thus, the material density of the model has a distinct effect on its equivalent density. The extreme values of the equivalent density, which enhanced the visible-range absorption, are listed in Table 1.

#### 3.1.4. Relationship between Equivalent Thermal Conductivity and Optical Absorption

Thermal conductivity is an important physical parameter for optical absorbers, with respect to infrared scene generation and photoacoustic effects. Optical absorbers with high thermal conductivities exhibit fast heat dissipation and high-sensitivity time-domain temperature responses. Optical absorbers with low thermal conductivities can generate high-temperature radiation signals at relatively low incident laser powers. The relationship between the equivalent thermal conductivity of the model and its filling factor can be estimated from the classical cheese model [22], which is as follows:

(12) $$k_{eff}=k_{0}\;\cdot \;\varepsilon +k_{m}\;\cdot \;(1-\varepsilon ),$$

where *k_eff_* is the equivalent thermal conductivity of the model; *k*_0_ is the thermal conductivity of the material used; *k_m_* is the thermal conductivity of the medium, and $\varepsilon =\gamma^{2/3}$ represents the two-dimensional (2D) porosity in the direction of heat conduction. After combining Equations (7) and (10), we obtain the following equation:

(13) $$A=c+C_{1}\;\cdot \;[{\left(\frac{k_{eff}}{k_{0}}\right)}^{\frac{3}{2}}+C_{2}\;\cdot \;{\left(\frac{k_{eff}}{k_{0}}\right)}^{3}]\;\cdot \;e^{-d\;/\;C_{3}},$$

**Figure 8:**
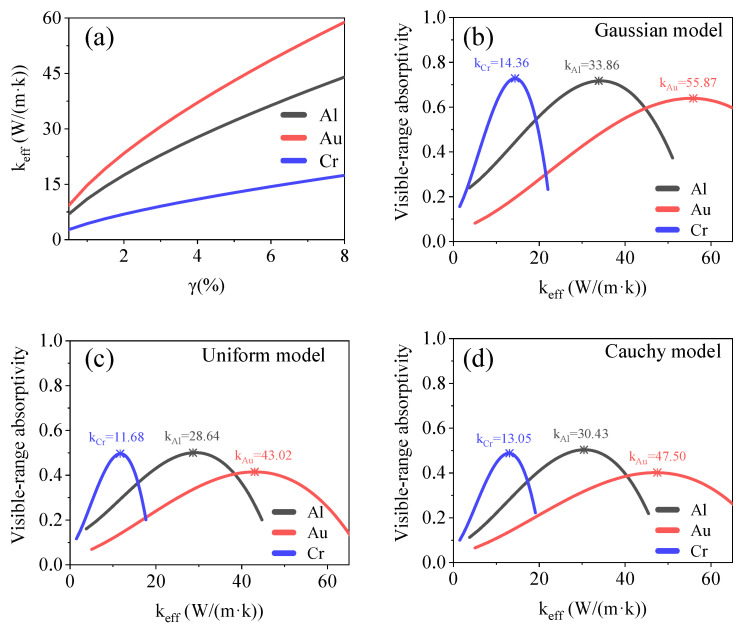
(**a**) Relationship between filling factor and equivalent thermal conductivity of model used. (**b**–**d**) Relationship between equivalent thermal conductivity and visible-range absorptivity for Gaussian, uniform, and Cauchy models. Equivalent thermal conductivity of model with maximum absorptivity is marked in the figures.

Figure 8 shows the relationship between the filling factor, equivalent thermal conductivity, and visible-range absorptivity for the Gaussian, uniform, and Cauchy models. The extreme values of the equivalent thermal conductivity, which enhanced the visible-range absorption, are listed in Table 1. Thus, Au nanoaggregate structures are more suitable as optical absorbers that must exhibit efficient absorption and fast heat dissipation. However, Cr nanoaggregate structures allow for efficient energy capture and accumulation.

#### 3.1.5. Relationship between Equivalent Volumetric Heat Capacity and Optical Absorption

**Figure 9:**
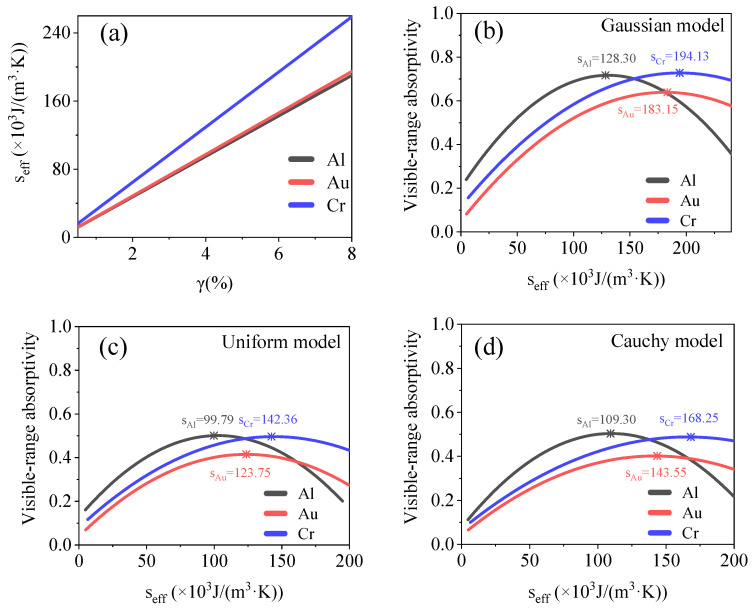
(**a**) Relationship between filling factor and equivalent volumetric heat capacity of model. (**b**–**d**) Relationship between equivalent volumetric heat capacity and visible-range absorptivity of Gaussian, uniform, and Cauchy models. Equivalent volumetric heat capacity of model with maximum absorptivity is marked in the figures.

The specific heat capacity affects the time-domain temperature response rate of optical absorbers. High optical absorption and low specific heat capacity are essential material properties for ensuring a strong photoacoustic effect [10,23]. Similar to the case for the equivalent density, the relationship between the filling factor and equivalent volume heat capacity of the model can be described as follows:

(14) $$s_{eff}=s_{0}\;\cdot \;\gamma +s_{m}\;\cdot \;(1-\gamma ),$$

(15) $$A=c+C_{1}\;\cdot \;[\frac{s_{eff}}{s_{0}}+C_{2}\;\cdot \;{\left(\frac{s_{eff}}{s_{0}}\right)}^{2}]\;\cdot \;e^{-d\;/\;C_{3}},$$

where *s_eff_* is the equivalent volumetric heat capacity of the model; *s*_0_ is the volumetric heat capacity of the material used, and *s_m_* is the volumetric heat capacity of the medium. The equivalent volumetric heat capacities of the Al and Au models were almost equal, while that of the Cr model was slightly higher, as shown in Figure 9. The extreme values of the equivalent volumetric heat capacity, which enhanced the visible-range absorption, are listed in Table 1. Because its absorptivity is higher, the Al nanoaggregate structure is more suitable as an optical absorber that shows a low volumetric heat capacity and high-sensitivity time-domain temperature response.

### 3.2. Model Validation

#### 3.2.1. Sample Characterization

**Figure 10:**
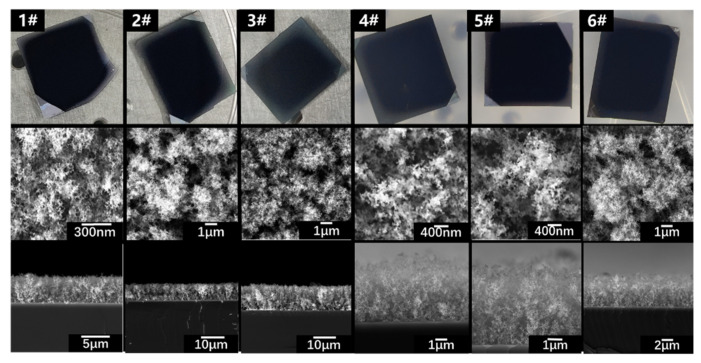
Photographs and SEM images of nanoaggregate samples. Samples #1–3 were formed on 500 μm Si substrates. Samples #4–6 were formed on 300 nm PI layer; PI layer was fabricated by spin-coating.

We fabricated six different samples with nanoaggregate-structured layers. Photographs and SEM images of the samples are shown in Figure 10. The thickness and 2D porosity of the layers of the nanochain aggregates were measured. The filling factor of the samples was calculated using the expression $\varepsilon =\gamma^{2\;/\;3}$. The structural parameters of the samples are listed in Table 2.

**Figure 11:**
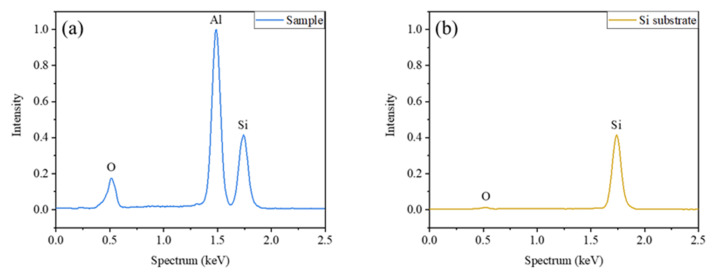
EDS results of the sample and Si substrate. (**a**) Results of the sample are normalized based on Al peak intensity, and the relative weight ratio of O, Al and Si is 0.175:1:0.416. (**b**) Results of Si substrate are normalized based on the Si weight ratio in the sample (0.416), and the relative weight ratio of O and Si is 0.011:0.416.

Energy dispersive spectroscopy (EDS, Ultim Extreme, Oxford, UK) detection is performed on the sample and Si substrate to quantitatively analyze the oxide content in Al nano aggregates. Figure 11a shows the types (O, Al and Si) and the relative weight ratio (0.175:1:0.416) of the elements contained in the sample. According to the relative atomic mass of each element, the atomic ratio of O, Al and Si is calculated as 0.0109:0.037:0.0149. Since EDS detection is carried out in vacuum, the O element is derived from aluminum oxide and silicon oxide. The Al element is derived from aluminum and oxide in the nano aggregates, and the Si element is derived from silicon and oxide in the substrate. Figure 11b shows the types (O and Si) and the relative weight ratio (0.011:0.416) of the elements contained in the substrate. Results of the substrate are normalized based on the Si weight ratio in the sample (0.416). The atomic ratio of O and Si is calculated as 0.0007:0.0149. The O element in the substrate is derived only from silicon oxide. Therefore, it can be calculated that 93.6% of the O element in the sample is derived from aluminum oxide and 6.4% from silicon oxide. In addition, 81.5% of the Al element is derived from aluminum; 18.5% from aluminum oxide. The molecular ratio of aluminum to its oxide is calculated as 8.81:1.

**Figure 12:**
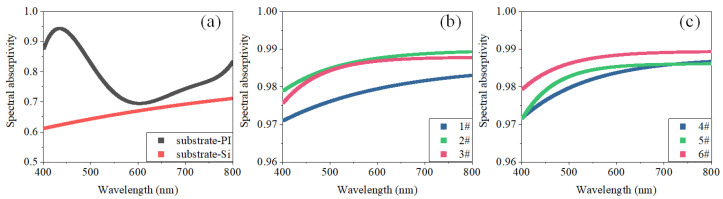
Measured spectral absorption curves of various substrates and nanoaggregate samples. (**a**) Measured spectral absorption curves of Si and PI substrates. (**b**) Measured spectral absorption curves of samples #1–3. (**c**) Measured spectral absorption curves of samples #4–6.

The spectral absorption curves of the samples in the 400–800 nm band were measured using a dual optical path ultraviolet–visible (UV–vis) spectrophotometer (TU-1901, Persee, Beijing, China). Two standard reflectance plates were used for correction. All the samples were supported by a 500 μm Si wafer, which was completely opaque in the visible range. The spectral absorptivities of the samples were calculated using Equation (7). The spectral absorption curves of the Si substrates, PI substrates, and samples are shown in Figure 12. The root mean square error (RMSE) between the measured and fitted curves was less than 0.7%, as shown in Table 3. The absorptivities of the samples were higher than 0.97 in the 400–800 nm band.

#### 3.2.2. Analysis of Calculation and Experimental Results

**Figure 13:**
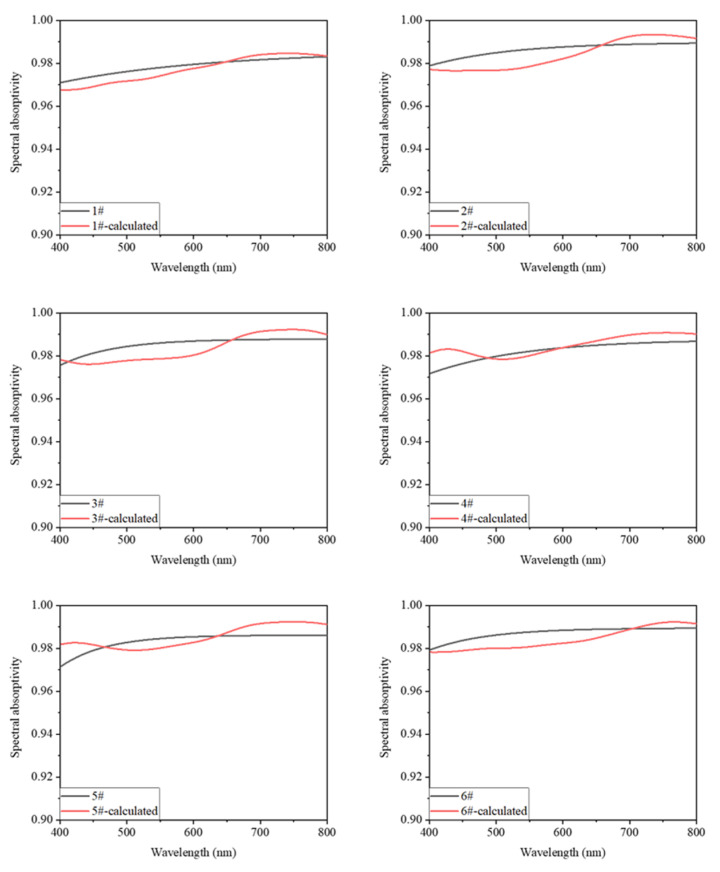
Spectral absorption curves determined based on weighted Gaussian, uniform, and Cauchy distribution models and those obtained experimentally.

We constructed a physical model of the nanoaggregate samples based on the structural parameters listed in Table 2. The spectral reflectivity and absorptivity of the substrate used were included in the model as the boundary conditions. The weights of the three distribution functions in the aggregation model were adjusted to optimize the calculation results. The values of *W*_G_, *W*_U_, and *W*_C_ were set at 0.6, 0.2, and 0.2, respectively. A comparison of the calculation and experimental results is shown in Figure 13. The Spearman correlation coefficient was used to evaluate the correlation between the calculation and measurement curves; the values obtained are listed in Table 4.

**Figure 14:**
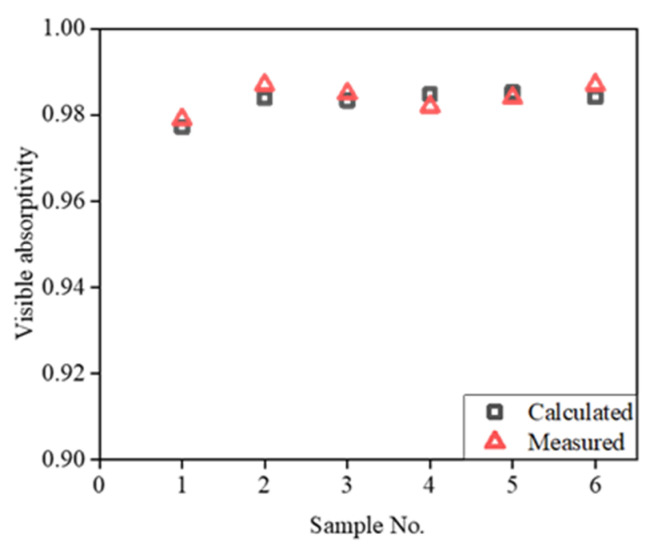
Calculated and measured values of visible-range absorptivity of various samples.

To evaluate the accuracy of the model described by Equation (9), we compared the calculated values of the visible-range absorptivity with the measured ones, as shown in Figure 14. The relative errors are presented in Table 4.

The calculated spectral absorption curves of the samples are in good agreement with the experimental curves, with the Spearman correlation coefficients being higher than 0.82. Within the range of values used for the structural parameters of the samples, the relative errors between the calculated visible-range absorptivities and those determined experimentally were less than 0.3%. This confirmed that the model was accurate.

### 3.3. Discussion

The model of nanoaggregate structure presented here is only a preliminary model, which is mainly established and verified for Al. Due to the limitation of the experimental conditions, the samples have a relatively small range of fill factor and thickness compared to the range of the presented model predictions. Therefore, the accuracy of the model is only verified in a relatively small range. We will study and improve the fabrication method to expand the range of sample thickness and filling factor in subsequent research. In addition, the model should be further improved to make it universal. The general form of nanoaggregate structure can be applied to metal absorbers obtained by various processes. This model is suitable for the study of surface absorption of various optical sensors, photothermal effect and photovoltaic, as well as terahertz generation and detection.

## 4. Conclusions

In this study, the structures of randomly distributed nanochain aggregates on silicon substrates were modeled, and the model was evaluated. The relationship between the structural parameters (thickness and filling factor), equivalent physical parameters (density, heat capacity, and thermal conductivity), and visible-range absorptivity of the model were established and analyzed. All the above-mentioned parameters exhibited extreme values, which enhanced the visible-range absorptivity. The accuracy of the model was verified experimentally. The following conclusions were drawn based on the results obtained.

(1) The visible-range absorptivity of the structure was quadratically related to the filling factor. The filling factor affects the motion path of incident photons and, thus, the efficiency of capturing photons. An extremely sparse or dense distribution of nanochains leads to a reduction in the model absorptivity.
(2) The visible-range absorptivity of the modeled structure is exponentially related to its thickness. The critical thickness of the Gaussian model is much smaller than those of the uniform and Cauchy models because of the higher photon-capturing efficiency of the former. The maximum visible-range absorptivities of the three models are similar, indicating that the aggregate form of the nanochains has a negligible effect on the surface reflection of the model.
(3) The visible-range absorptivity of the modeled structure is quadratically related to its equivalent density. The Al nanochain aggregate structure is more suitable as an optical absorber that exhibits a low density and high visible-range absorption.
(4) The visible-range absorptivity of the modeled structure is also related to its equivalent thermal conductivity. The Au nanochain aggregate structure allows for efficient optical absorption and fast heat dissipation. Meanwhile, the Cr nanoaggregate structure allows for efficient energy capture and accumulation.
(5) Finally, the visible-range absorptivity of the modeled structure is quadratically related to its equivalent volumetric heat capacity. The Al nanoaggregate structure is more suitable as an optical absorber with a low volumetric heat capacity and high-sensitivity time-domain temperature response.

Actual nanochain aggregate samples were fabricated by reducing the kinetic energy of the deposited Al nanoparticle clusters. The visible-range spectral absorption curves of the fabricated samples were measured using a Fourier spectrometer. The Spearman correlation coefficients for the calculated spectral absorption curves and those measured experimentally were higher than 0.82; this confirmed the accuracy of the model. In addition, the relative errors between the calculated visible-range absorptivities and the measured values were less than 0.3%; this confirmed that the model is suitable for calculating the absorptivity.

## Figures and Tables

**Table 1:** Relationships between model parameters and visible-range absorptivity.

Parameter	Type of Relationship Curve	Material of the Models	Extreme Values for Gaussian, Uniform and Cauchy Models
Filling factor (%)	Quadratic	Al	*γ*_G_ = 5.4, *γ*_U_ = 4.4, *γ*_C_ = 4.6
		Au	*γ*_G_ = 7.4, *γ*_U_ = 5.0, *γ*_C_ = 5.6
		Cr	*γ*_G_ = 6.0, *γ*_U_ = 4.4, *γ*_C_ = 5.2
Thickness (μm)	Exponential	Al	*d*_G_ = 21, *d*_U_ = 29, *d*_C_ = 36
		Au	*d*_G_ = 47, *d*_U_ = 49, *d*_C_ = 59
		Cr	*d*_G_ = 37, *d*_U_ = 42, *d*_C_ = 43
Density (×10^3^ kg m^−3^)	Quadratic	Al	*ρ*_G_ = 0.146, *ρ*_U_ = 0.113, *ρ*_C_ = 0.124
		Au	*ρ*_G_ = 1.430, *ρ*_U_ = 0.996, *ρ*_C_ = 1.121
		Cr	*ρ*_G_ = 0.431, *ρ*_U_ = 0.316, *ρ*_C_ = 0.374
Thermal conductivity (W m^−1^ K^−1^))	Cubic	Al	*k*_G_ = 33.86, *k*_U_ = 28.64, *k*_C_ = 30.43
		Au	*k*_G_ = 55.87, *k*_U_ = 43.02, *k*_C_ = 47.50
		Cr	*k*_G_ = 14.36, *k*_U_ = 11.68, *k*_C_ = 13.05
Volumetric heat capacity (×10^3^ J m^−3^ K^−1^)	Quadratic	Al	*s*_G_ = 128.3, *s*_U_ = 99.79, *s*_C_ = 109.3
		Au	*s*_G_ = 183.15, *s*_U_ = 123.75, *s*_C_ = 143.55
		Cr	*s*_G_ = 194.13, *s*_U_ = 123.75, *s*_C_ = 143.55

**Table 2:** Structural parameters of nanoaggregate samples.

Number	1#	2#	3#	4#	5#	6#
Thickness of the absorbed layer (μm)	4.78	7.34	8.37	4.83	5.07	5.56
Filling factor (%)	6.1	5.1	4.1	5.9	6.3	6.8
Substrate thickness and material	500 μm Si			300 nm PI + 500 μm Si		

**Table 3:** Statistics of measured error.

Object	RMSE between Measured and Fitted Curves
Si	0.0044
1#	0.0008
2#	0.0001
3#	0.0007
PI	0.0071
4#	0.0002
5#	0.0007
6#	0.0001

**Table 4:** Comparison of calculation and measurement results.

Object	Spearman Correlation Coefficient of Spectral Absorption Curves	Relative Error of Visible Absorptivity
1#	0.976	0.0018
2#	0.954	0.0031
3#	0.962	0.0017
4#	0.846	0.0028
5#	0.822	0.0013
6#	0.996	0.0029

## Data Availability

Not applicable.
